# Specific isoforms of the ubiquitin ligase gene *WWP2* are targets of osteoarthritis genetic risk via a differentially methylated DNA sequence

**DOI:** 10.1186/s13075-024-03315-8

**Published:** 2024-04-03

**Authors:** Jack B. Roberts, Olivia L.G. Boldvig, Guillaume Aubourg, S. Tanishq Kanchenapally, David J. Deehan, Sarah J. Rice, John Loughlin

**Affiliations:** 1 Biosciences Institute, Newcastle University, International Centre for Life, Newcastle upon Tyne, NE1 3BZ UK; 2 Freeman Hospital, Newcastle University Teaching Hospitals NHS Trust, Newcastle upon Tyne, UK

**Keywords:** Genetics, Epigenetics, DNA methylation, Epigenome editing, *WWP2*, miR-140

## Abstract

**Background:**

Transitioning from a genetic association signal to an effector gene and a targetable molecular mechanism requires the application of functional fine-mapping tools such as reporter assays and genome editing. In this report, we undertook such studies on the osteoarthritis (OA) risk that is marked by single nucleotide polymorphism (SNP) rs34195470 (A > G). The OA risk-conferring G allele of this SNP associates with increased DNA methylation (DNAm) at two CpG dinucleotides within *WWP2*. This gene encodes a ubiquitin ligase and is the host gene of microRNA-140 (miR-140). *WWP2* and miR-140 are both regulators of TGFβ signaling.

**Methods:**

Nucleic acids were extracted from adult OA (arthroplasty) and foetal cartilage. Samples were genotyped and DNAm quantified by pyrosequencing at the two CpGs plus 14 flanking CpGs. CpGs were tested for transcriptional regulatory effects using a chondrocyte cell line and reporter gene assay. DNAm was altered using epigenetic editing, with the impact on gene expression determined using RT-qPCR. *In silico* analysis complemented laboratory experiments.

**Results:**

rs34195470 genotype associates with differential methylation at 14 of the 16 CpGs in OA cartilage, forming a methylation quantitative trait locus (mQTL). The mQTL is less pronounced in foetal cartilage (5/16 CpGs). The reporter assay revealed that the CpGs reside within a transcriptional regulator. Epigenetic editing to increase their DNAm resulted in altered expression of the full-length and N-terminal transcript isoforms of *WWP2*. No changes in expression were observed for the C-terminal isoform of *WWP2* or for miR-140.

**Conclusions:**

As far as we are aware, this is the first experimental demonstration of an OA association signal targeting specific transcript isoforms of a gene. The *WWP2* isoforms encode proteins with varying substrate specificities for the components of the TGFβ signaling pathway. Future analysis should focus on the substrates regulated by the two *WWP2* isoforms that are the targets of this genetic risk.

**Supplementary Information:**

The online version contains supplementary material available at 10.1186/s13075-024-03315-8.

## Background

The regulatory RNA microRNA-140 (miR-140) and its host gene, *WWP2*, are abundantly expressed in articular cartilage, with their knockout in mice adversely affecting chondrogenesis and cartilage tissue homeostasis, resulting in osteoarthritis (OA)-like phenotypes [1–6]. A double knockout has a more severe phenotype than a single knockout, implying that miR-140 and WWP2 protein function cooperatively in the generation and maintenance of cartilage [3].

miR-140 is a known regulator of proteins implicated in the TGFβ signaling pathway, for example the SMAD signal transducers [1]. This pathway controls the synthesis of the extracellular matrix (ECM) and in the musculoskeletal system it plays an essential role in the health of articulating joints [7, 8]. WWP2 protein is a member of the NEDD4 E3 family of ligases that mediate the transfer of ubiquitin to protein substrates, which can alter their cellular localization or result in their proteasomal degradation [9–12]. By regulating steady-state levels of target proteins, the ubiquitin-proteasomal pathway controls many cellular processes, including the cell cycle, apoptosis, and immune response [9–12]. Full-length (FL) WWP2 contains three functional components; an amino-terminal C2 domain that binds to protein substrate, four WW domains (WW1-WW4) that confer substrate specificity, and a carboxyl HECT domain that accepts ubiquitin before transferring it to the substrate [10]. In addition to WWP2-FL, there are two other common isoforms: WWP2-N, which lacks WW domains 2–4 and the HECT domain, and WWP2-C, which lacks WW domains 1–3 and the C2 domain [10]. Like miR-140, WWP2 also regulates TGFβ signaling: WWP2-FL controls availability of SMAD2/3, with WWP2-N interacting with WWP2-FL to modulate this action [10, 13], whilst WWP2-C also interacts with WWP2-FL, to control availability of SMAD7, a TGFβ signal inhibitor [10, 13, 14]. The levels of isoforms FL, N and C therefore fine-tune TGFβ signaling, influencing cellular phenotype [9, 10, 13–15].

In 2018, a genome-wide association study (GWAS) meta-analysis of Icelandic and UK data sets reported a knee OA association signal mapping to the *WWP2*/miR-140 locus [16]. The lead DNA variant, rs34195470 (A > G), is located within an intron of *WWP2*, with allele G conferring increased OA risk (odds ratio (OR) 1.07). In 2019, a GWAS of the UK Biobank and arcOGEN datasets reported association close to the *WWP2*/miR-140 locus, this time with knee or hip OA [17]. The lead variant, rs6499244 (T > A), is located upstream of *WWP2* and within *NFAT5*, with allele A conferring increased OA risk (OR 1.06). The pair-wise linkage disequilibrium (LD) between the two variants is modest in European ancestry cohorts but sufficiently high to suggest that they are marking the same association signal: r^2^ and D’ of 0.22 and 0.51 respectively. In 2021, the largest yet OA GWAS, encompassing 826,690 individuals, also reported rs34195470 and rs6499244 as associated variants [18]. rs34195470 was highlighted as the lead one of the two, and a potential causal variant, with association *P*-value of 3.13 × 10^− 13^ [18]. Interestingly, in all three of the OA GWAS publications, association signals were also reported to genes encoding members of the TGFβ pathway: extracellular ligands, transmembrane receptors, intracellular signaling molecules, and transcription factors [16–18]. These results imply that this pathway, and regulators of it, such as *WWP2* and miR-140, are targets of OA genetic risk.

None of the known polymorphisms in LD with rs34195470 alter the amino acid coding sequence of *WWP2* or the sequence of miR-140, implying that the genetic signal mapping to this locus mediates its effect by modulating gene expression. This is the case for most variants that are associated with complex polygenic diseases such as OA, with a high proportion of such risk-conferring variants residing in enhancer regulatory elements located in intergenic or intronic regions [19–21]. In 2019, an allelic expression imbalance (AEI) study of protein-coding genes reported several *WWP2* SNPs that associated with AEI of the gene in OA cartilage [22]. The most significant imbalance was at rs1052429 (G > A), with a relative increased expression of allele A (56% of this allele versus 44% for allele G), equivalent to an A/G ratio of 1.27 [22]. rs1052429 has pairwise r^2^ and D’ of 0.41 and 0.91 with rs34195470, which means that the OA risk-conferring allele G of rs34195470 nearly always occurs on a haplotype containing allele A of rs1052429. The OA risk allele of rs34195470 therefore associates with increased *WWP2* expression in cartilage.

Using genome-wide DNA methylation (DNAm) array data created from the cartilage of patients who had undergone joint arthroplasty surgery, we previously reported that genotype at rs34195470 and rs6499244 associated with the methylation levels of CpG dinucleotides located within *WWP2*, forming methylation quantitative trait loci (mQTLs) [23, 24]. Both variants associated with methylation of the same CpGs, cg26736200 and cg26661922, and for each CpG the OA risk-conferring alleles of rs34195470 and rs6499244 associated with increased methylation [23, 24]. An additional cartilage mQTL at the *WWP2* locus (cg02712546) has also been identified, located 17.3Kb from cg26736200 and cg26661922 [25]. cg26736200 and cg26661922 are 115 bp apart, less than 4Kb from rs34195470, and reside in a region marked as having enhancer activity [23]. This implies that DNAm may be an intermediate between the genetic risk signal and changes in gene expression, with the causal variant altering methylation of the enhancer which then alters expression of the target gene. DNAm as a functional intermediate of genetic risk is a relatively common phenomenon [26–28] and we have previously used associating DNAm signatures to experimentally characterize OA risk loci and identify target genes of risk-conferring alleles [29–34]. These experiments have been conducted on cartilage from arthroplasty patients and on cartilage from human foetal samples, the latter used to assess whether OA risk alleles have functional impact during joint development [33, 34].

Based on the highly compelling GWAS data and the essential role of *WWP2* and miR-140 in cartilage health, rs34195470 is a particularly interesting OA genetic risk signal. Using cartilage from arthroplasty patients and foetal samples, a chondrocyte cell line, and a range of molecular and *in silico* approaches, we analysed the rs34195470 mQTL to assess if it linked the risk signal with target gene/s expression.

## Methods

### Cartilage samples

Cartilage was obtained from 54 patients undergoing arthroplasty surgery at the Newcastle upon Tyne NHS Foundation Trust hospitals for primary hip OA (n = 25) or primary knee OA (n = 29). The NHS Health Research Authority granted ethical approval for sample collection with donors providing verbal and written consent (REC reference number 19/LO/0389). Nucleic acids were extracted as previously described [29–31]. Forty-eight foetal cartilage samples taken from the ends of the femur and tibia were provided by the Human Developmental Biology Resource (project 200363). Nucleic acids were extracted as previously described [33]. Additional file 1 contains further details regarding the OA patients and foetal samples.

### Genotyping

Allelic quantification pyrosequencing assays were designed using the PyroMark Assay Design 2.0 software (Qiagen) and oligonucleotide primers were ordered from Integrated DNA Technologies (IDT). Genomic DNA harbouring the regions containing rs34195470 and rs1052429 were amplified using the PyroMark PCR kit (Qiagen) to manufacturer protocol and genotype determined using the PyroMark Q24 Advanced system (Qiagen). Primer sequences available in Additional file 2.

### DNA methylation quantification

Genomic DNA (500ng) was bisulphite converted using the EZ DNA Methylation kit (Zymo Research). CpGs, including cg26736200 and cg26661922, were PCR amplified using three methylation quantification pyrosequencing assays (Additional file 2). Assays were designed using the PyroMark Assay Design 2.0 software (Qiagen) with primers ordered from IDT. DNAm levels were quantified at the CpGs using the PyroMark Q24 Advanced system (Qiagen). Analyses were performed in duplicate and replicate values with > 5% difference were excluded.

### AEI analysis

Complementary DNA (cDNA) was reverse transcribed from 500ng of OA patient cartilage RNA using SuperScript IV (Invitrogen). cDNA and pyrosequencing were then used to determine the relative ratio of A:G in nine patients heterozygous at rs1052429. Samples were analysed in triplicate. Replicate values with > 5% differences were excluded. Allelic expression in cDNA was normalised to genomic DNA for each patient. Primer sequences available in Additional file 2.

### In silico analysis

The UCSC genome browser (hg19) [35] was used to visualise the genomic region encompassing rs34195470, cg26736200 and cg26661922, miR-140, and the three main characterized transcript isoforms of *WWP2*: full-length (*WWP2*-FL, RefSeq ID NM_007014), N-terminal (*WWP2*-N, RefSeq ID NM_001270455) and C-terminal (*WWP2*-C, RefSeq ID NM_199424). Human chromatin state data was mined from the ROADMAP Epigenomics Project [36] to identify regulatory function across the *WWP2*/miR-140 locus, focusing on H1 human embryonic stem cell line-derived (H1-derived) mesenchymal stem/stromal cells (MSCs; E006) and chondrocytes differentiated from bone marrow-derived stem/stromal cells (E049). To investigate the presence of long-range chromatin interactions at the CpG region, Capture Hi-C data in H1-derived MSCs was mapped using the 3D Genome Browser [37]. This technology aids in the delineation of functional and spatial regulatory interactions by mapping regions that are in close physical proximity in 3D but linearly distant in 2D. To assess if rs34195470 or the CpGs were in open or closed chromatin, ATAC-sequencing data generated from the cartilage chondrocytes of five OA knee patients, five OA hip patients, six foetal knee samples, and six foetal hip samples was investigated [33] (Gene Expression Omnibus (GEO; https://www.ncbi.nlm.nih.gov/geo/) accession number GSE214394). JASPAR [38] was used to search for transcription factors (TFs) predicted to bind at or close to CpGs, using a predicted score value > 500, equivalent to *P* < 1 × 10^− 5^. The expression of TFs was assessed using RNA-sequencing data generated from the hip cartilage of ten OA patients [39] (GEO accession number GSE111358).

### Reporter gene assay

A 282 bp region encompassing the CpGs was amplified using genomic DNA and the insert was cloned into the pCpG-free-Basic-Lucia plasmid (Invivogen) to assess promoter function, and into the pCpG-free-Promoter-Lucia plasmid (Invivogen) to assess enhancer function (primer sequences available in Additional file 3). Successful cloning was determined by Sanger sequencing (Source Bioscience). Plasmids were methylated or mock-methylated using *M.SssI* (New England Biolabs). Tc28a2 immortalised chondrocytes [40] were seeded at a density of 5000 cells/well in 96-well plates and incubated for 24 h. Then, cells were transfected with 100ng insert DNA-vector constructs and 10ng pGL3-promoter-luciferase plasmid (Promega) using Lipofectamine 2000 (Invitrogen) and incubated. After 24 h, cells were lysed, and luminescence measured using the Dual Luciferase Reporter Assay system (Promega). Twelve biological replicates were performed per plasmid.

### Epigenetic modulation using catalytically dead Cas9 (dCas9)

Using the IDT design tool, five CRISPR guide RNA (gRNA) sequences (gRNAs 1–5) were designed to target the region harbouring the CpGs, with a non-targeting gRNA used as a control (Additional file 3). Sequences were synthesised as single-stranded DNA primers (IDT), annealed and cloned into the catalytically dead Cas9 (dCas9)-DNA methyltransferase 3a (DNMT3a)-enhanced green fluorescent protein (EGFP) plasmid (Addgene, #71666) as previously described [31]. 5 µg gRNA-dCas9-DNMT3a-EGFP was nucleofected (Lonza Amaxa 4D-Nucleofector) into 10^6^ Tc28a2 cells and seeded in 6-well plates, with successful transfection confirmed after 24 h using EGFP visualisation (Axiovision, Zeiss). Cells were harvested after reaching 80% confluence. Nucleic acids were extracted using the Norgen DNA/RNA kit (Norgen Bio-Tek). Seven biological replicates were performed per gRNA. Gene expression was measured by reverse transcription quantitative PCR (RT-qPCR) using pre-designed TaqMan or universal probe library assays (Additional file 4) and the 2^−ΔΔCt^ method [41]. Isoform-specific assays were used to measure *WWP2*-FL, *WWP2*-N, and *WWP2*-C. RNA (1 µg) was reverse transcribed using SuperScript IV (Invitrogen), diluted 1:20 and expression was normalised to the housekeeping genes *18S*, *GAPDH* and *HPRT1*. For miR-140, assays specific to each of its two strands (miR-140-5p and miR-140-3p) were used. RNA (100ng) was reverse transcribed using MultiScribe (Thermo Fisher Scientific), diluted 1:6 and expression was normalised to the housekeeping small nuclear RNA *U6*.

### Statistical analysis

For graphical representations of DNAm data, methylation status was plotted in the form of β-values, ranging from 0 (no methylation) to 1 (100% methylation). For statistical analysis of methylation data, β-values were converted to M-values [42]. In mQTL analysis, simple linear regression was used to assess the relationship between CpG methylation and rs34195470 genotype. Mann-Whitney U test was used to calculate *P*-values when comparing DNAm levels irrespective of genotype. Wilcoxon matched-pairs signed rank test was used to calculate *P*-values in AEI analysis. For Lucia reporter assays, *P*-values were calculated using Mann-Whitney U test with Holm-Šídák adjustment to account for multiple comparisons. Paired *t*-test was used to calculate *P*-values for changes in gene expression following epigenetic modulation, with Benjamini-Hochberg adjustment to account for multiple comparisons. Tests were performed in GraphPad Prism.

## Results

### Replication of the mQTL and AEI analysis

**Fig. 1:**
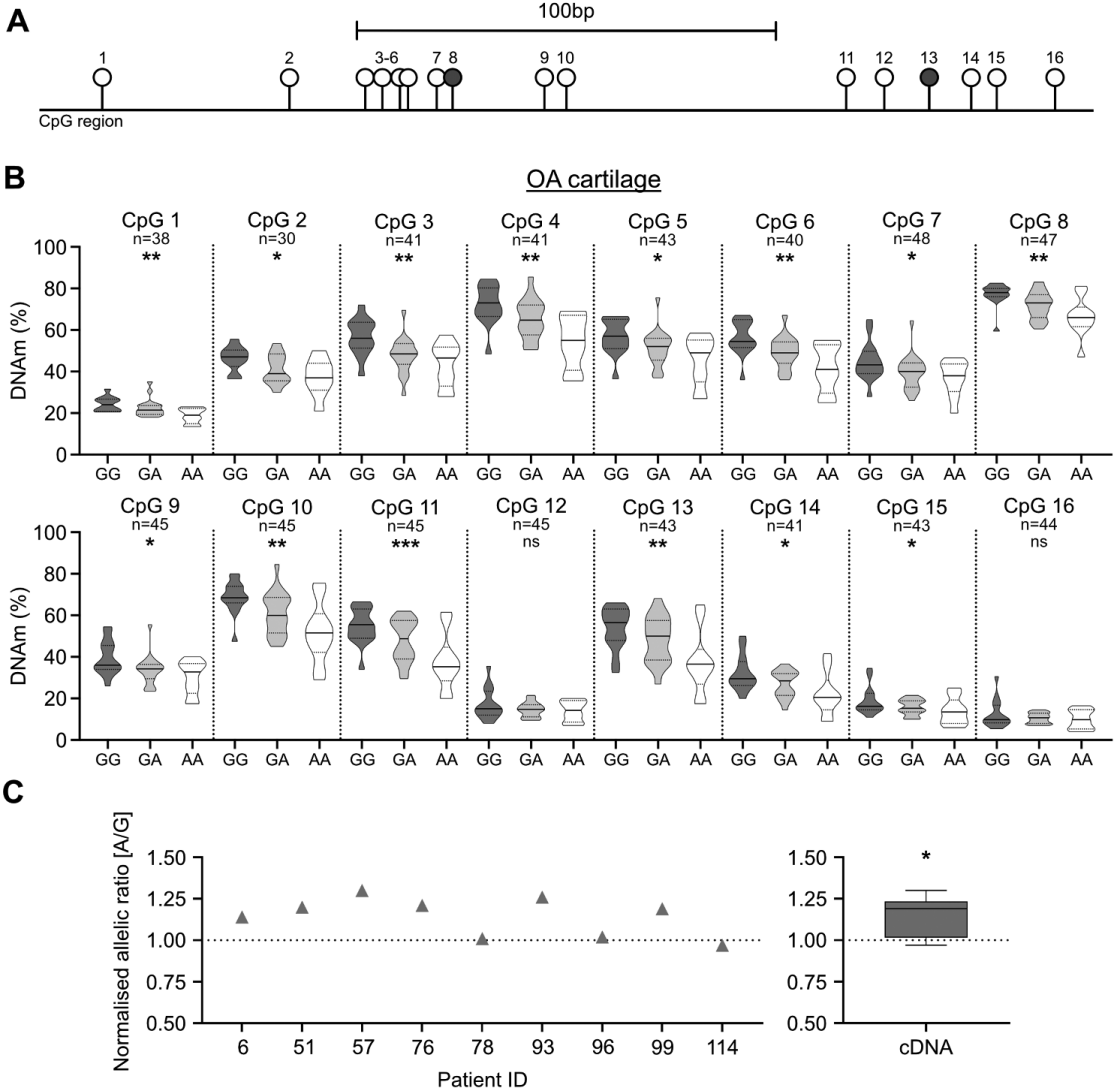
mQTL and AEI analysis of cartilage DNA and RNA from OA arthroplasty patients. **A** Schematic representation of the 16 CpGs analysed in this study across a 228 bp region. cg26736200 (CpG8) and cg26661922 (CpG13) are highlighted. **B** DNAm levels at the 16 CpGs stratified by rs34195470 genotype (GG, GA, AA). Methylation data is in the form of β-values ranging from 0 (no methylation) to 1 (complete methylation) and expressed as a percentage. In the violin plots, solid and dashed horizontal lines represent the median and interquartile range. Difference in numbers (n) due to variable number of patient samples per CpG passing quality control. *P*-values calculated by linear regression. * = *P* < 0.05; ** = *P* < 0.01; *** = *P* < 0.001; ns = not significant (*P* > 0.05). **C** Left, allelic (A/G) ratios in cartilage samples from nine OA patients heterozygous for *WWP2* transcript SNP rs1052429 (A = OA risk allele). cDNA values are plotted as grey triangles, representing the mean of three replicates normalised to DNA values (represented by dotted line). Right, mean cDNA values for all samples represented as a box plot normalised to their corresponding DNA values, as above. Line inside the box represents the median, the box shows the interquartile range, the whiskers show the minimum and maximum values. *P*-value calculated by Wilcoxon matched-pairs signed rank test. * = *P* < 0.05

We repeated the rs34195470 mQTL analysis of cg26736200 and cg26661922 in an independent cohort of cartilage DNA from OA arthroplasty patients, simultaneously expanding the study to encompass nearby CpGs (Fig. 1 and Additional file 5). We generated data on 16 CpGs in total: cg26736200 (CpG8 in Fig. 1A) and cg26661922 (CpG13); the four CpGs in-between these two (CpG9-CpG12), the three CpGs immediately downstream of cg26661922 (CpG14-CpG16) and 7 of the 8 CpGs immediately upstream of cg26736200 (CpG1-CpG7; we were unable to generate data for a single CpG located in-between CpG1 and CpG2). Fourteen of the 16 CpGs showed a significant association (*P* < 0.05) between DNAm levels and genotype at rs34195470, with the OA risk-conferring G allele of rs34195470 associating with increased methylation (Fig. 1B). This replicated our original discovery [23, 24] and highlighted the presence of a differentially methylated region (DMR) extending over 200 bp. Stratification by sex or joint revealed that the mQTL was not restricted to a particular stratum (Additional file 6). The OA arthroplasty patients range in age from 41-93 years. The DNAm levels for CpGs 11 and 13 showed a modest (r^2^ ≤ 0.1) but significant (*P* ≤ 0.05) reduction with age (Additional file 7). Stratification of the DNAm data by sex or joint site irrespective of rs34195470 genotype (Additional file 8, panels A and B) revealed only one significant difference in methylation levels, with CpG12 being slightly more methylated in hip compared to knee cartilage. Significant AEI (*P* < 0.05) was observed in nine OA cartilage samples heterozygous at rs1052429, with a mean A/G ratio of 1.15 (Fig. 1C and Additional file 9). This is equivalent to the A/G ratio of 1.27 previously reported [22] and confirms that the OA risk-conferring allele associates with increased expression of *WWP2*.

### The mQTL is detectable in foetal cartilage DNA

**Fig. 2:**
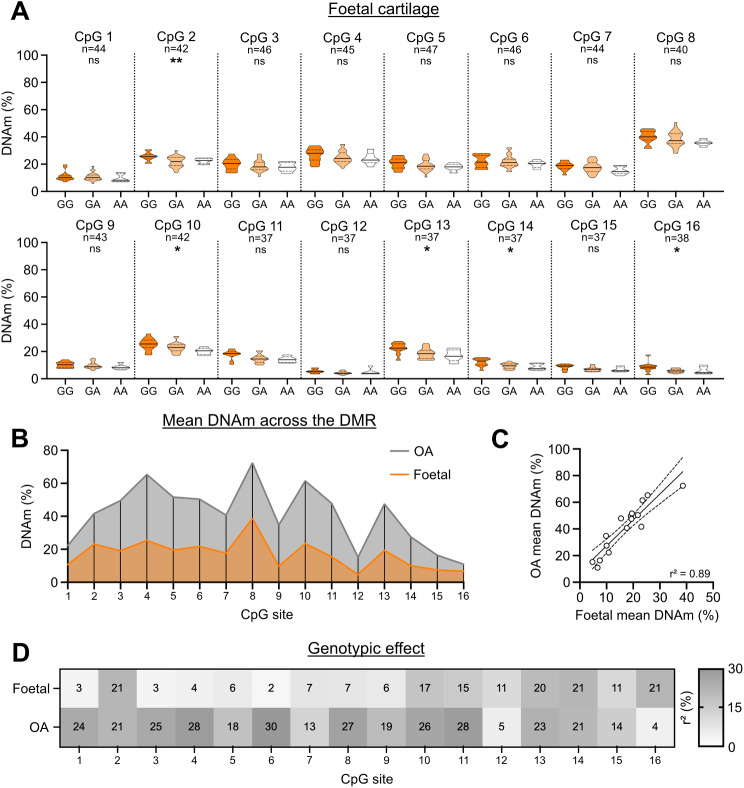
mQTL analysis of cartilage DNA from foetal samples. **A** DNAm levels at the 16 CpGs stratified by rs34195470 genotype. Methylation data is in the form of β-values ranging from 0 (no methylation) to 1 (complete methylation) and expressed as a percentage. In the violin plots, solid and dashed horizontal lines represent the median and interquartile range. Difference in numbers (n) due to variable number of patient samples per CpG passing quality control. *P*-values calculated by linear regression analysis. * = *P* < 0.05; ** = *P* < 0.01; ns = not significant (*P* > 0.05). **B** Plot of the mean cartilage DNAm values for each CpG for the OA arthroplasty patients (grey) and for the foetal samples (orange). **C** Comparison of the mean DNAm values for each CpG between the OA arthroplasty patients and the foetal samples. r^2^ value determined by linear regression. Line of best fit and 95% confidence intervals shown. **D** Heatmap showing the influence of the rs34195470 genotype on DNAm levels at the 16 CpGs in the foetal samples and in the OA arthroplasty patients. r^2^ values determined by linear regression and expressed as a percentage

We have reported that for several OA SNPs the association with CpG methylation observed in arthroplasty cartilage also occurs in foetal cartilage, implying that a proportion of OA genetic risk may be functionally active during skeletogenesis [33, 34]. We therefore assessed whether the rs34195470 mQTL was detectable in foetal cartilage. Five of the 16 CpGs showed a significant association (*P* < 0.05) between methylation and rs34195470 genotype, and as for the arthroplasty samples, the OA risk-conferring G allele associated with increased methylation in the significant CpGs (Fig. 2A and Additional file 5). For all 16 CpGs, the mean methylation levels were lower in foetal compared to OA arthroplasty (Fig. 2B), but with a strong positive correlation between the two cartilage tissues with an r^2^ of 0.89 (Fig. 2C). We used linear regression to calculate the percentage contribution of rs34195470 genotype to differences in DNAm in foetal and arthroplasty (Fig. 2D). The genotypic effect was larger in OA arthroplasty cartilage DNA for 12 of the 16 CpGs and was the same between the two tissues for 2 of the remaining 4 CpGs. There were no differences between females and males when the foetal DNAm data was stratified by sex irrespective of rs34195470 genotype (Additional file 8, panel C).

### The DMR acts as a methylation-sensitive regulator of gene expression

**Fig. 3:**
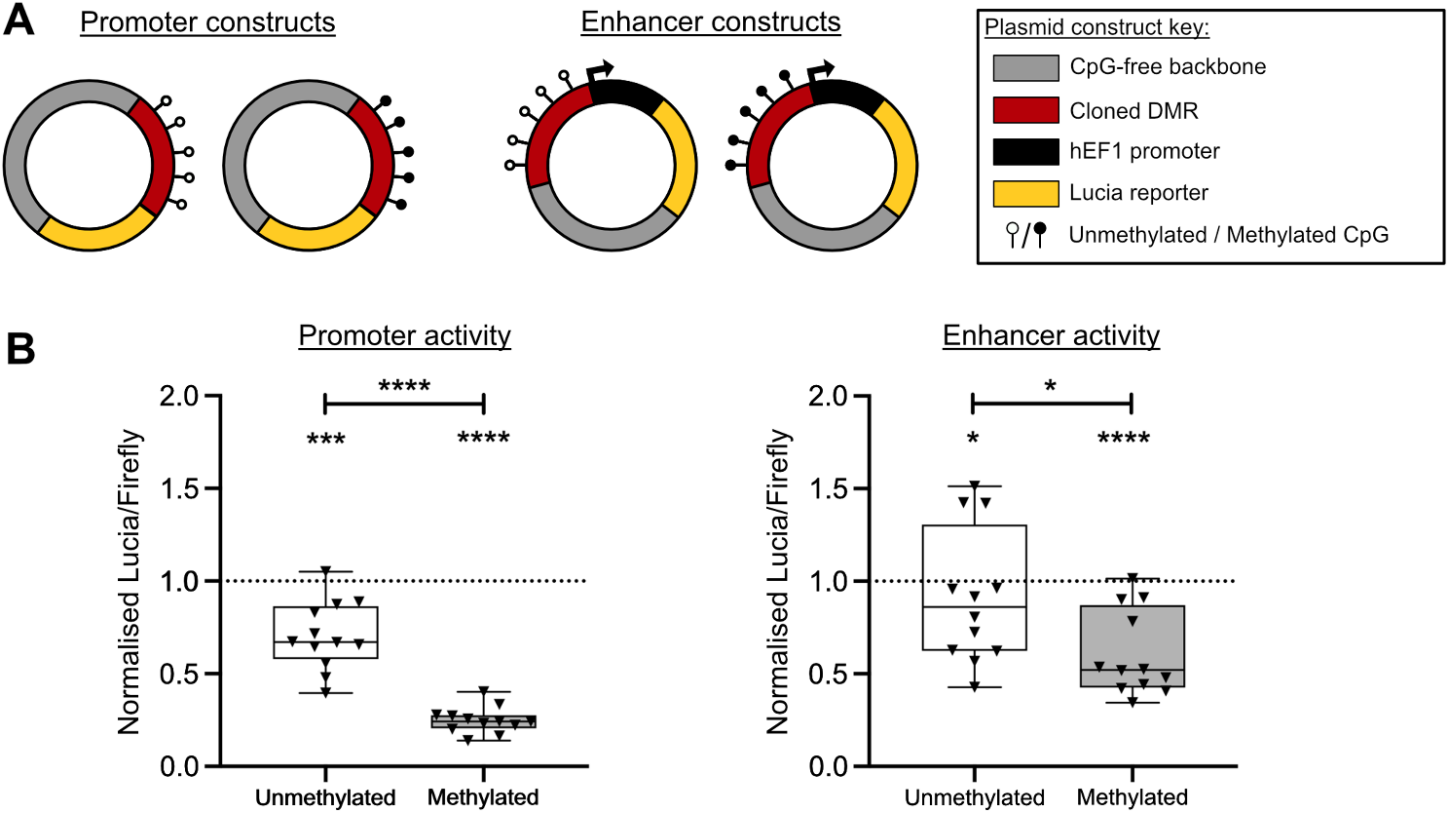
Investigation of the transcriptional regulatory function of the DMR in Tc28a2 chondrocytes. **A** Schematic representation of the promoter and enhancer plasmid vector constructs. **B** Lucia reporter assays assessing promoter or enhancer activity in the presence of construct containing the DMR in an unmethylated or methylated state. Values were normalised to those in empty vector control (dotted horizontal line). Black dots represent individual samples (12 replicates per group). Box plots show the median, 25^th^ and 75^th^ percentiles, and minimum and maximum values. *P*-values calculated by Mann Whitney U test with Holm-Šídák correction. * = *P* < 0.05; *** = *P* < 0.001; **** = *P* < 0.0001

To assess promoter and enhancer activity, and to study the effect of DNAm on these activities, the DMR was cloned into Lucia reporter gene vectors and transfected into Tc28a2 cells (Fig. 3 and Additional file 10). When cloned as a promoter, the unmethylated DMR repressed gene expression compared to empty vector control, with this repression increasing following methylation (Fig. 3B, left panel); *P* < 0.001 for unmethylated DMR versus control, *P* < 0.0001 for methylated DMR versus control, *P* < 0.0001 for unmethylated DMR versus methylated DMR. When cloned as an enhancer, the unmethylated DMR repressed gene expression compared to empty vector control, with this repression increasing following methylation (Fig. 3B, right panel); *P* < 0.05 for unmethylated DMR versus control, *P* < 0.0001 for methylated DMR versus control, *P* < 0.05 for unmethylated DMR versus methylated DMR. This data implies the DMR is a methylation-sensitive regulator of gene expression.

### Increased DNA methylation at the DMR increases expression of ***WWP2*** in an isoform-specific manner

**Fig. 4:**
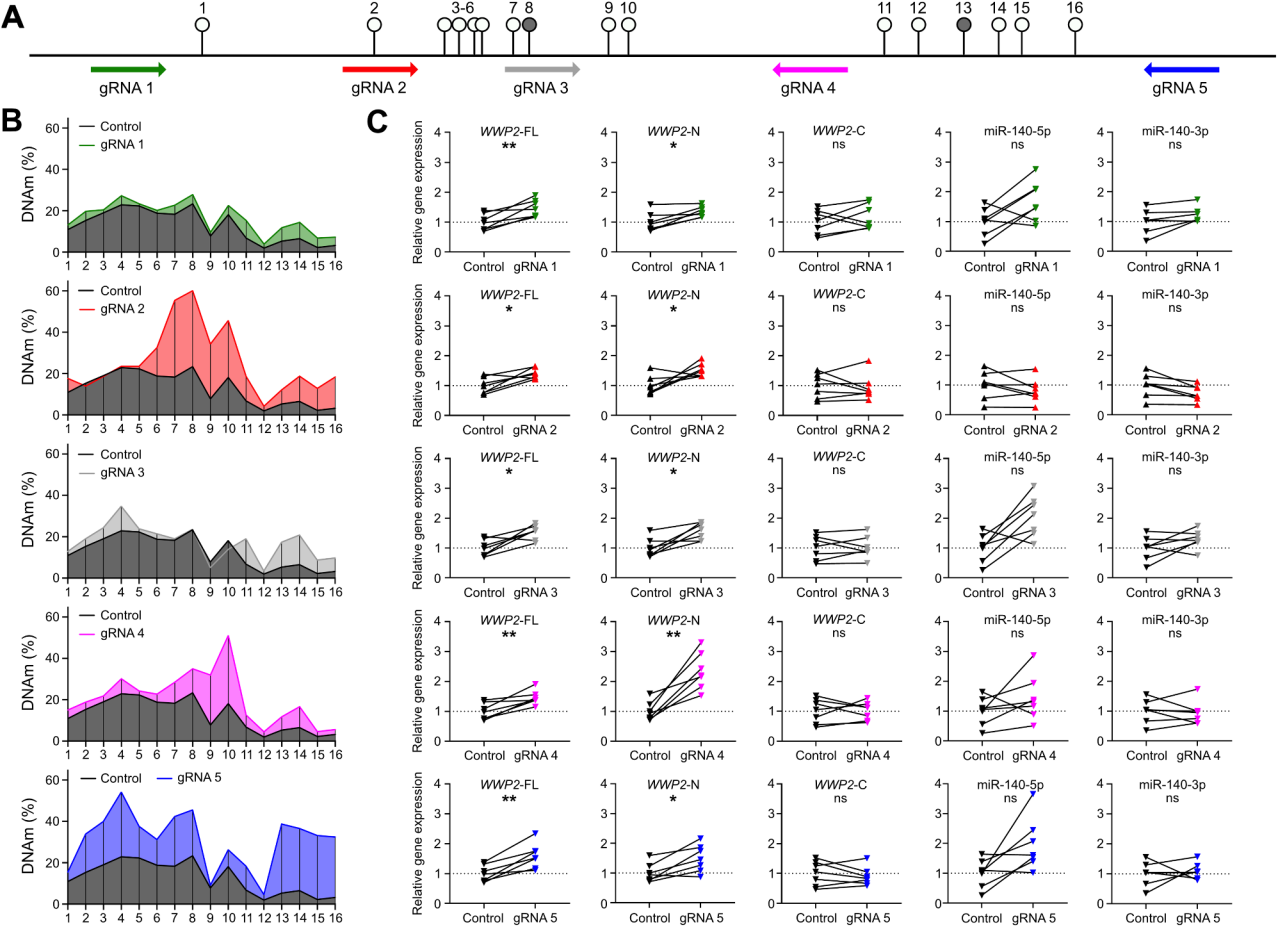
Epigenetic modulation of the DMR in Tc28a2 chondrocytes. **A** Schematic representation of the 16 CpGs and of the five gRNAs (arrow pointing left, antisense strand; arrow pointing right, sense strand). cg26736200 (CpG8) and cg26661922 (CpG13) are highlighted. **B** Mean DNAm levels (%) of the 16 CpGs following expression of dCas9-DNMT3a protein in control (black), with non-targeting gRNA, or in samples with a targeting gRNA (coloured). Seven replicates for control and for each targeting gRNA. **C** Relative expression of the three *WWP2* transcripts and of the two miR-140 strands following epigenetic editing with gRNAs. Values were normalised to non-targeting gRNA. *P*-values were calculated using a paired *t*-test with Benjamini-Hochberg correction. * = *P* < 0.05; ** = *P* < 0.01; ns = not significant (*P* > 0.05)

We next performed epigenetic editing of the DMR in Tc28a2 chondrocytes and measured the effect upon expression of the three common isoforms of *WWP2* (*WWP2*-FL, *WWP2*-N, *WWP2*-C) and on the two miR-140 strands (miR-140-5p and miR-140-3p) (Fig. 4 and Additional files 11 and 12). Tc28a2 cells are heterozygous (GA) at rs34195470 and moderately hypomethylated at the DMR, with DNAm levels ranging from 1.9% to 23.4% across the 16 CpGs (Fig. 4B, control data, in black). We therefore methylated the DMR using transiently expressing gRNA-dCas9-DNMT3a constructs. Five gRNAs (gRNA 1–5) were designed to target across the DMR in both orientations of the genome (Fig. 4A). An increase in DNAm (> 5%) at a minimum of three CpGs was observed for all gRNAs, with gRNA5 increasing methylation for 13 of 16 CpGs (Fig. 4B, in blue). The largest overall increase in DNAm was 37.7% at CpG7 by gRNA2 (Fig. 4B, in red). Mean percentage increases in DNAm levels for all CpGs ranged from 4.0% (gRNA1) to 18.8% (gRNA5). For all five gRNAs, we observed a significant increase in expression (all *P*-values < 0.05) of the *WWP2*-FL and *WWP2*-N isoforms in response to the increase in DMR methylation (Fig. 4C). No significant changes in expression (all *P*-values > 0.05) for any gRNA were seen for *WWP2*-C, miR-140-5p or miR-140-3p.

### The DMR resides in an enhancer that physically interacts with the promoter of the ***WWP2***-FL and ***WWP2***-N isoforms

**Fig. 5:**
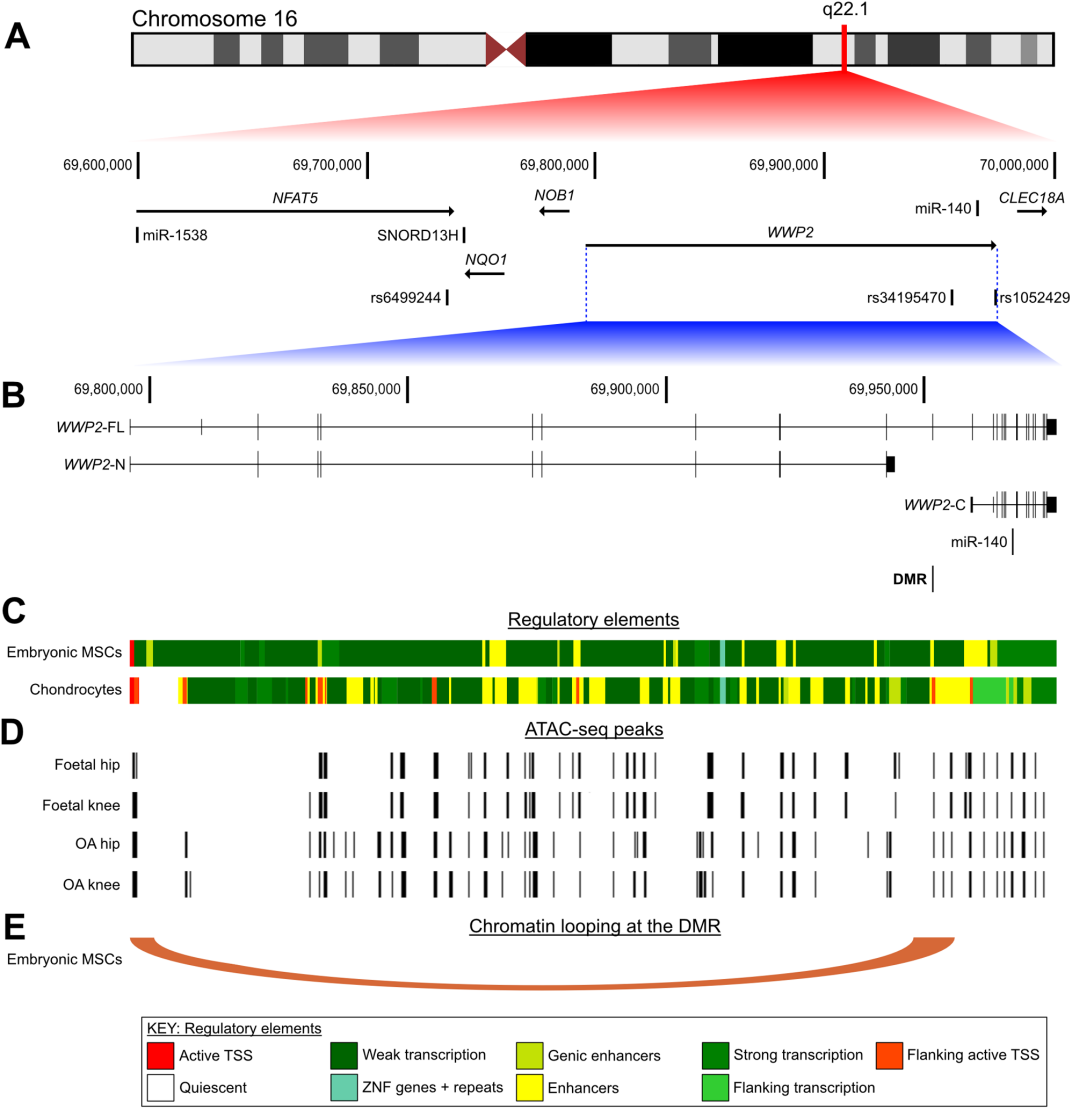
*In-silico* analysis of the region harbouring *WWP2*, rs34195470 and the DMR. **A** Schematic representation of chromosome 16q22.1, expanding to show key genomic features proximal to *WWP2* including other genes, microRNAs and small nucleolar RNAs (snoRNAs). Three SNPs are marked underneath this: rs6499244, rs34195470 and rs1052429. **B** The genomic region is further expanded to show the gene structure of the *WWP2* isoforms *WWP2*-FL, *WWP2*-N and *WWP2*-C. Horizontal lines represent introns, full height vertical bars represent exons, and half-height vertical bars represent 5’ and 3’ UTRs. The location of miR-140 and the DMR are also shown. Coordinates from UCSC hg19. **C** Chromatin regulatory state data from ROADMAP for H1-derived embryonic MSCs and chondrocytes. Colours corresponding to the different regulatory elements shown at bottom of figure. **D** ATAC-sequencing peaks generated from foetal hip, foetal knee, OA hip, and OA knee chondrocytes. Open chromatin regions marked by vertical black lines. **E** Capture Hi-C chromatin interactions identified at the DMR in H1-derived embryonic MSCs using the 3D Genome Browser, represented as a loop with the flat ends of the loop spanning the width of the interacting regions

The *WWP2*-FL and *WWP2*-N mRNAs are derived from the same promoter, with *WWP2*-N arising through a splicing event involving intron retention and a premature termination codon [9] (Fig. 5B). *WWP2*-C mRNA and miR-140 derive from the utilization of a separate promoter, located within an intron of *WWP2* [9] (Fig. 5B). The DMR spans an exon-intron boundary and rs34195470 resides within the intron shared with the *WWP2*-C/miR-140 promoter (Fig. 5B). The DMR region is marked as an enhancer in MSCs and flanking an active transcription start site (TSS) in chondrocytes, whilst rs34195470 resides within an enhancer in chondrocytes (Fig. 5C). The DMR is within a region of open chromatin in foetal and OA arthroplasty chondrocytes (Fig. 5D). Capture Hi-C of MSCs reveals a long-range chromatin interaction between the region containing the DMR and rs34195470 and the *WWP2*-FL and *WWP2*-N promoter (Fig. 5E).

### The DMR is predicted to bind transcription factors that are expressed in cartilage

**Fig. 6:**
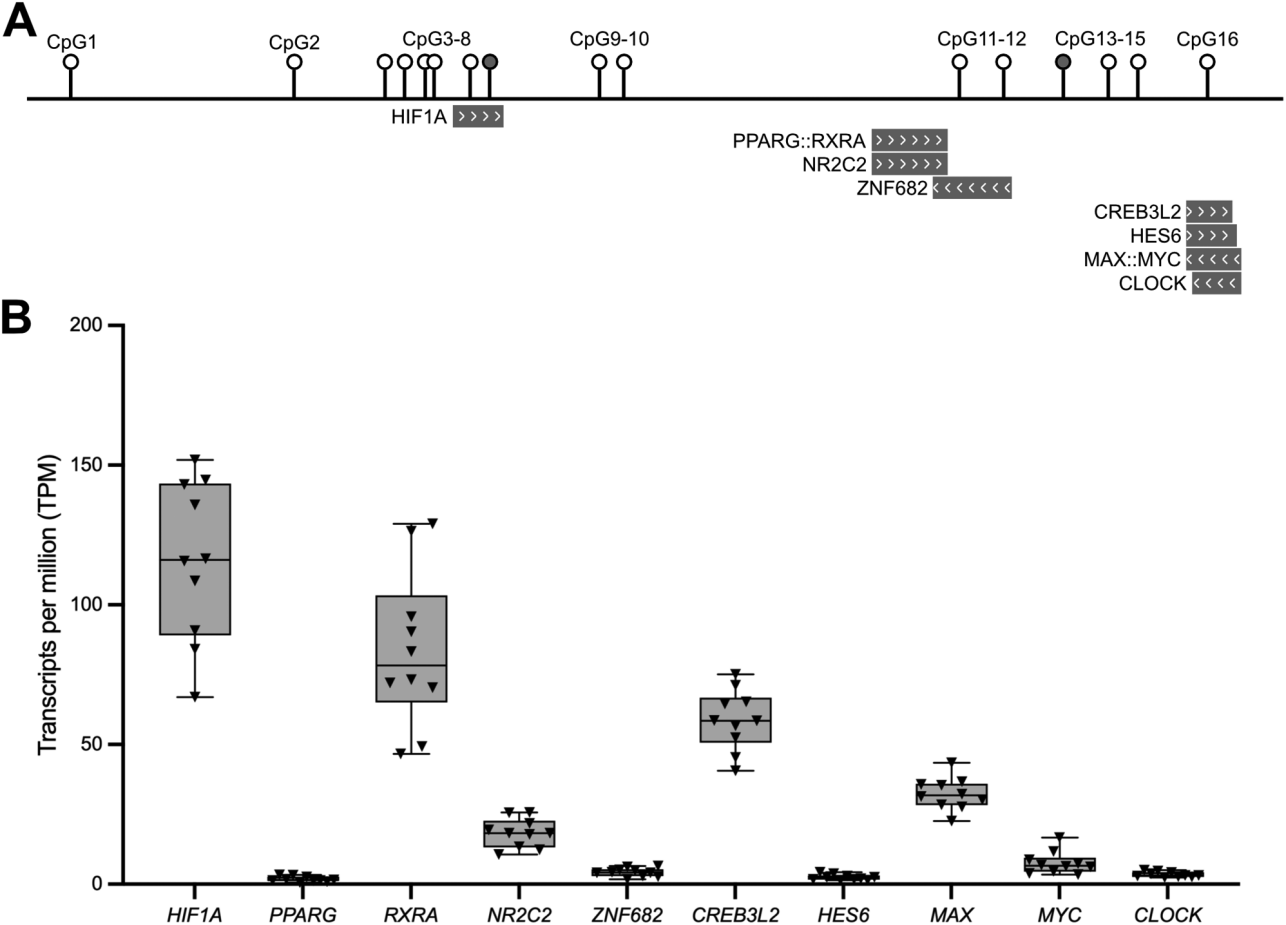
Transcription factors (TFs) predicted to bind at the DMR. **A** Schematic representation of the 16 CpGs and of 10 TFs predicted to bind at or close to the CpGs. cg26736200 (CpG8) and cg26661922 (CpG13) are highlighted. The TFs are marked by grey rectangles with the direction of the arrows within the rectangles indicating the DNA strand the TF is predicted to bind to (arrows pointing to the left = antisense strand, arrows pointing to the right = sense strand). TF heterodimers are denoted by a double colon (::) between two TFs. **B** Expression levels (TPM, transcripts per million) of the TFs in cartilage chondrocytes from 10 OA patients. Triangles represent individual samples. Box plots show the median, 25^th^ and 75^th^ percentiles, and minimum and maximum values

Epigenetic modulation and *in silico* analysis indicated that the DMR is a regulator of gene expression via interaction with the promoter of *WWP2*-FL and *WWP2*-N. If this were the case, the DMR would be expected to bind transcription factors (TFs) [28]. A search of JASPAR [38] identified ten TFs predicted to bind at or near the DMR CpGs (Fig. 6A). The genes for three of these TFs are abundantly expressed in cartilage, with transcripts per million (TPM) values > 50: *HIF1A* (encoding hypoxia-inducible factor-1α), *RXRA* (encoding retinoic acid receptor α) and *CREB3L2* (encoding cAMP-responsive element-binding protein 3-like protein 2) (Fig. 6B).

## Discussion

In our initial experiment, we replicated the rs34195470 mQTL in OA arthroplasty cartilage DNA, with the OA risk-conferring G allele associating with increased DNAm. The DMR extended over 200 bp and was detectable in hip and knee cartilage. We also confirmed that the risk-conferring allele associates with increased *WWP2* expression in cartilage. Reporter assays showed that the DMR has transcriptional effects, acting as a methylation-sensitive repressor, whereas with epigenetic editing, increased DNAm resulted in increased *WWP2* expression. The discordancy between the reporter and editing experiments likely reflects the relatively artificial nature of a reporter assay which, unlike genome editing, investigates a piece of DNA isolated from its normal genomic and chromatin context. The epigenetic editing highlighted *WWP2*-FL and *WWP2*-N as the target transcripts of the DMR, with no significant effects on *WWP2*-C or miR-140 expression. The small relative fold changes observed in the expression of *WWP2*-FL and *WWP2*-N following epigenetic editing match the fold differences in allelic expression measured in our AEI analysis (mean rs1052429 A/G ratio of 1.15) and reflect the small effect sizes observed for most polygenic risk loci, contributing to disease when inherited with multiple other risk loci [24]. Our reporter gene and epigenetic editing experiments were performed on a cell line; future studies undertaken on primary cells, including patient chondrocytes, may provide further insight into the role of the DMR as a regulator of *WWP2*.

**Fig. 7:**
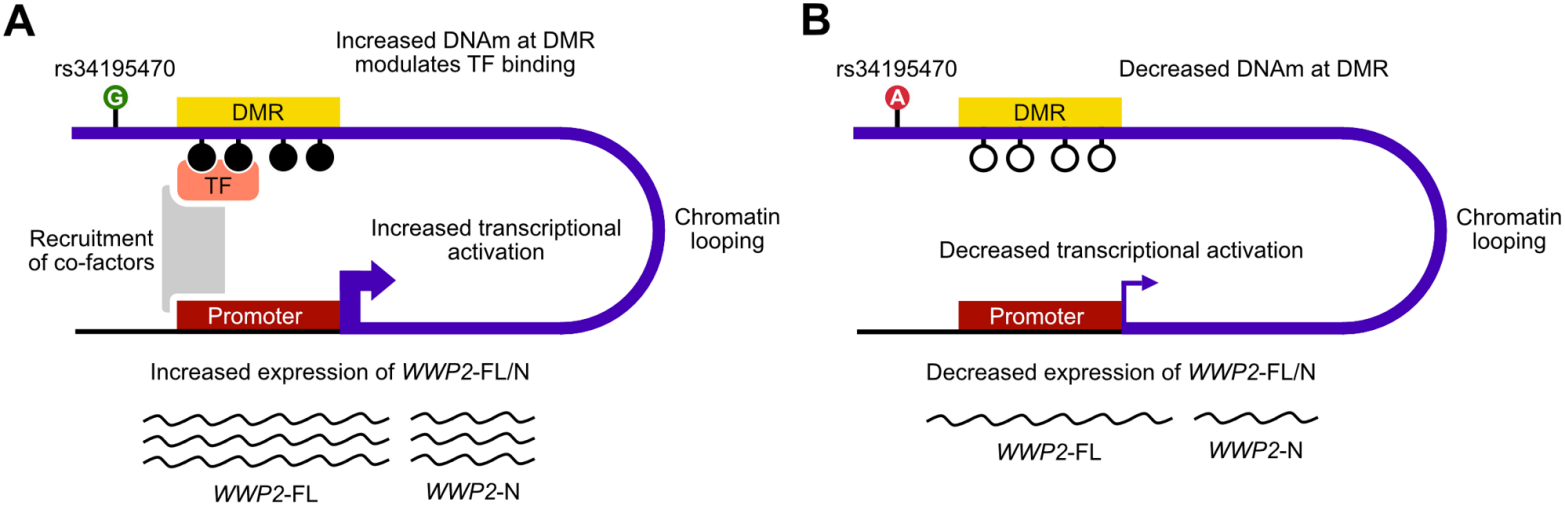
Proposed molecular model. **A** The DMR is part of a transcriptional activator that loops to the shared promoter of *WWP2*-FL and *WWP2*-N. In the presence of the OA-risk conferring allele G of rs34195470, the DMR is more highly methylated. This modulates binding of TFs to the DMR and increases its transcriptional activation potential via the recruitment of co-factors. This results in greater relative expression of *WWP2*-FL and *WWP2*-N. **B** In the presence of the OA non-risk conferring allele A of rs34195470, the DMR is less methylated. This results in decreased transcriptional activation potential and decreased relative expression of *WWP2*-FL and *WWP2*-N. Methylated CpGs are represented by filled circles; unmethylated CpGs are represented as unfilled circles. *WWP2*-FL mRNA is represented as longer wavy lines; *WWP2*-N mRNA is represented as shorter wavy lines

Using *in silico* data, we showed that the region of DNA harbouring the DMR is in open chromatin and loops to interact with an upstream region containing the promoter of *WWP2*-FL and *WWP2*-N. Finally, the DMR was predicted to bind TFs that are expressed in cartilage. Overall, we conclude that the rs34195470 association signal regulates the expression of *WWP2*-FL and *WWP2*-N via the epigenetic intermediate of differential DNAm. Our molecular model is as follows: (1) the DMR is part of a transcriptional activator; (2) it loops to the shared promoter of *WWP2*-FL and *WWP2*-N; (3) in the presence of OA-risk conferring allele G of rs34195470, the DMR is more highly methylated; (4) this modulates the binding of TFs to the DMR and increases its transcriptional activation potential; (5) this results in greater relative expression of *WWP2*-FL and *WWP2*-N (Fig. 7).

The DMR is in open chromatin in both foetal and adult chondrocytes and the mQTL was detectable in foetal cartilage DNA. However, the CpG methylation levels and the rs34195470 genotype effect were lower in foetal compared to adult cartilage DNA. An increase in CpG methylation over time is an active process mediated by DNMTs [28]. We conclude that the DMR is actively methylated after skeletogenesis and that the effect of genotype on methylation increases with age. The *WWP2* association signal may therefore be an example of antagonistic pleiotropy or genetic drift in OA, with the risk-conferring allele having a positive or neutral effect on joint formation but a negative effect on joint health as we age [43, 44].

In 2022 Kreitmaier and colleagues published a genome-wide methylation array and genotype array analysis of DNA extracted from the cartilage of OA patients [45]. They also reported the mQTL at cg26736200, using the *WWP2* intronic SNP rs61611907 located approximately 8.6Kb upstream of rs34195470 (pairwise r^2^ of 0.68 with rs34195470 in European ancestry cohorts). They applied Mendelian randomization to identify CpGs from the methylation array that have a potential causal role in OA and identified cg26736200 as one such site [45]. Our analysis of TFs that are predicted to bind at the DMR identified hypoxia-inducible factor-1α (HIF1A) as binding across CpG7 and CpG8/cg26736200. Of the ten TFs that we identified, the gene encoding HIF1A was the one with the highest level of expression in cartilage. This TF is a known regulator of chondrogenesis and of cartilage health [46, 47]. Tuerlings and colleagues also highlighted hypoxia as an important pathway in the activity of the rs34195470 association signal following upregulation of *WWP2*-FL expression in a 3D cartilage model [48]. These data imply that cg26736200, HIF1A, and hypoxia are key drivers of the association signal. Detailed investigation of their interaction is now merited.

## Conclusions

As far as we are aware, this is the first time that functional fine-mapping tools have been used to reveal that an OA association signal regulates the expression of specific isoforms of a gene. As noted in the introduction, WWP2 protein is a ubiquitin ligase that controls the cellular localization and steady-state levels of proteins [9–11]. WWP2-FL, WWP2-N and WWP2-C target different proteins, with the various components of the TGFβ signaling pathway being particularly well characterized substrates [10, 13]. It has been reported that WWP2-FL and WWP2-N, but not WWP2-C, contribute to cardiac fibrosis by interacting with and regulating the transcriptional activity of SMAD2 [15]. We hypothesize that a similar scenario is happening in OA, with the level of these two isoforms increased in carriers of the risk conferring allele G of rs34195470, resulting in altered interaction dynamics with substrate molecules and a negative impact on cartilage homeostasis. Any future clinical exploitation of this association signal should focus on the substrates targeted by WWP2-FL and WWP2-N, and on inhibitors targeting these two isoforms [49].

### Electronic supplementary material

Below is the link to the electronic supplementary material.

Supplementary Material 1

Supplementary Material 2

Supplementary Material 3

Supplementary Material 4

Supplementary Material 5

Supplementary Material 6

Supplementary Material 7

Supplementary Material 8

Supplementary Material 9

Supplementary Material 10

Supplementary Material 11

Supplementary Material 12

Supplementary Material 13

## Data Availability

Raw data is presented in Additional files 5, 9, 10, 11 and 12. Additional file 13 contains a summary of the statistical analyses performed.

## Abbreviations

AEI: allelic expression imbalance
CpG: Cytosine-Guanine dinucleotide
Cas9: CRISPR associated protein 9
CRISPR: Clustered regularly interspaced short palindromic repeats
dCas9: Deactivated (dead) Cas9
DNAm: DNA methylation
DMR: Differentially methylated region
DNMT3a: DNA (cytosine-5)-methyltransferase 3 A
gRNA: Guide RNA
GEO: Gene Expression Omnibus
mQTL: Methylation quantitative trait locus
NHS: National Health Service
OA: Osteoarthritis
RT-qPCR: Real-time quantitative polymerase chain reaction
SEM: Standard error of mean
TF: transcription factor
TPM: transcripts per million
UCSC: University of California Santa Cruz
UTR: Untranslated region
