# Supplementary figures and images for: Specific isoforms of the ubiquitin ligase gene WWP2 are targets of osteoarthritis genetic risk via a differentially methylated DNA sequence
Source: Arthritis Res Ther. 2024 Apr 3;26:78. doi: 10.1186/s13075-024-03315-8 (PMC10988806; doi:10.1186/s13075-024-03315-8)

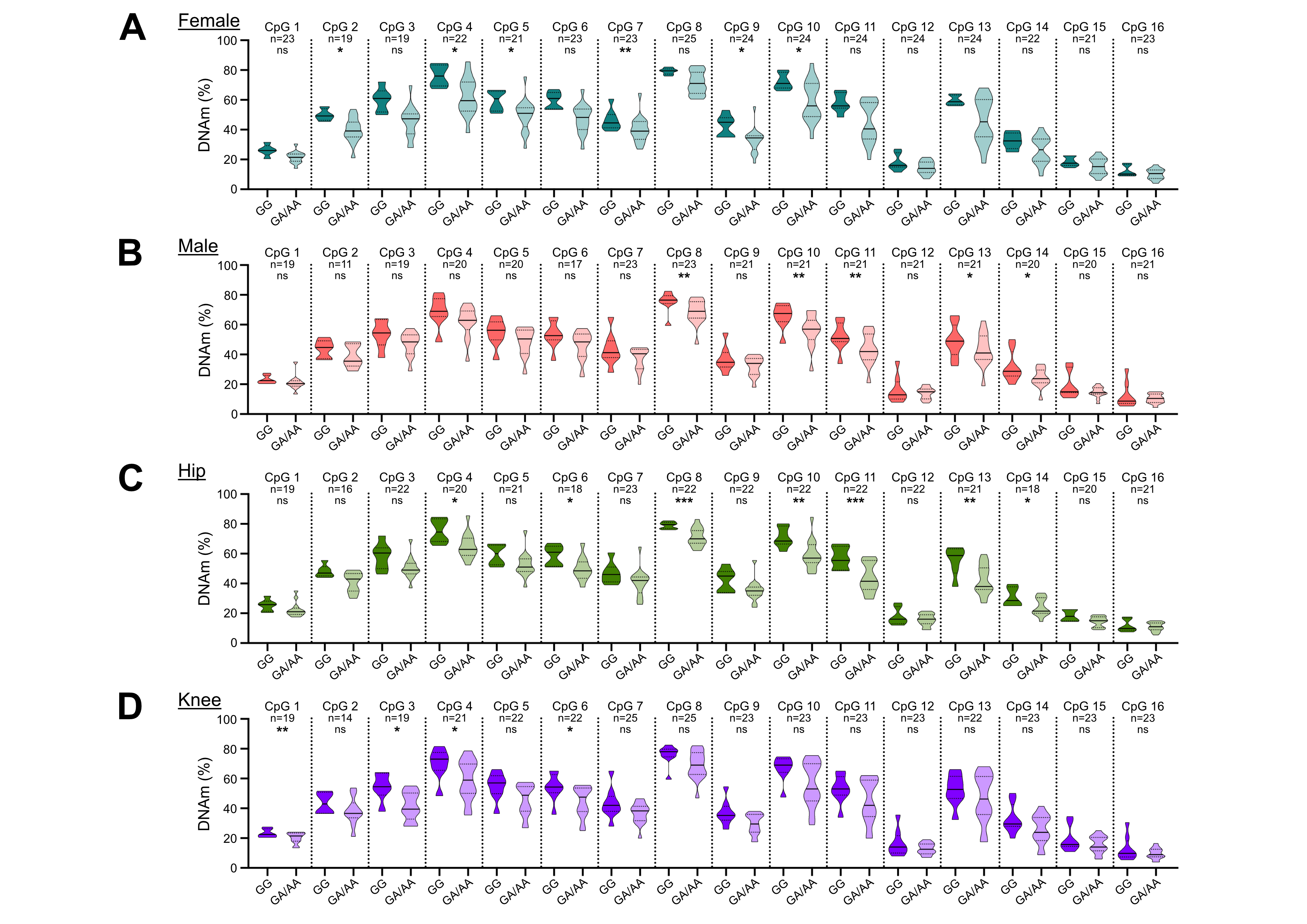

Supplement: Supplementary file 6 — Supplementary Material 6 [file 13075_2024_3315_MOESM6_ESM.tiff]

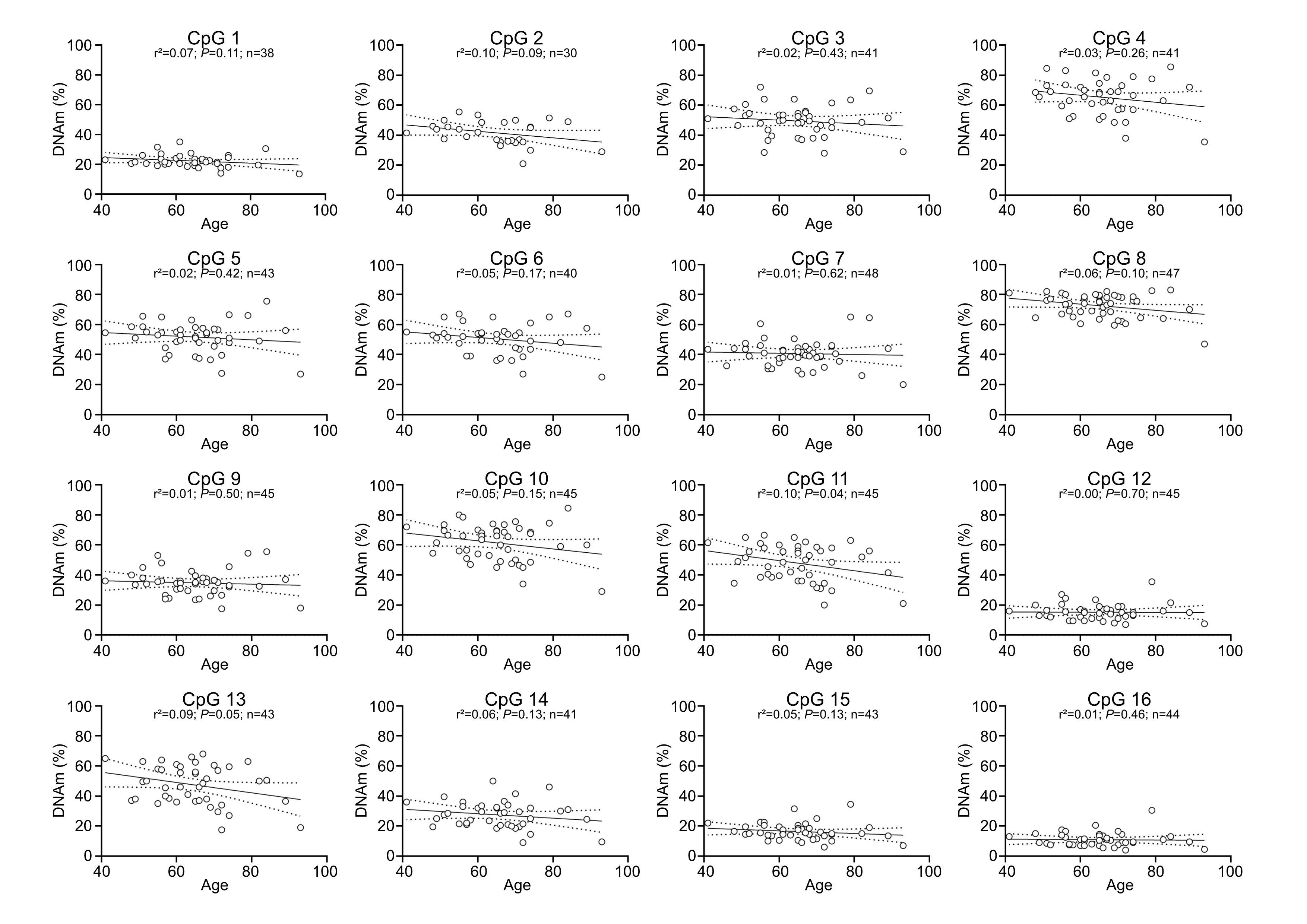

Supplement: Supplementary file 7 — Supplementary Material 7 [file 13075_2024_3315_MOESM7_ESM.tiff]

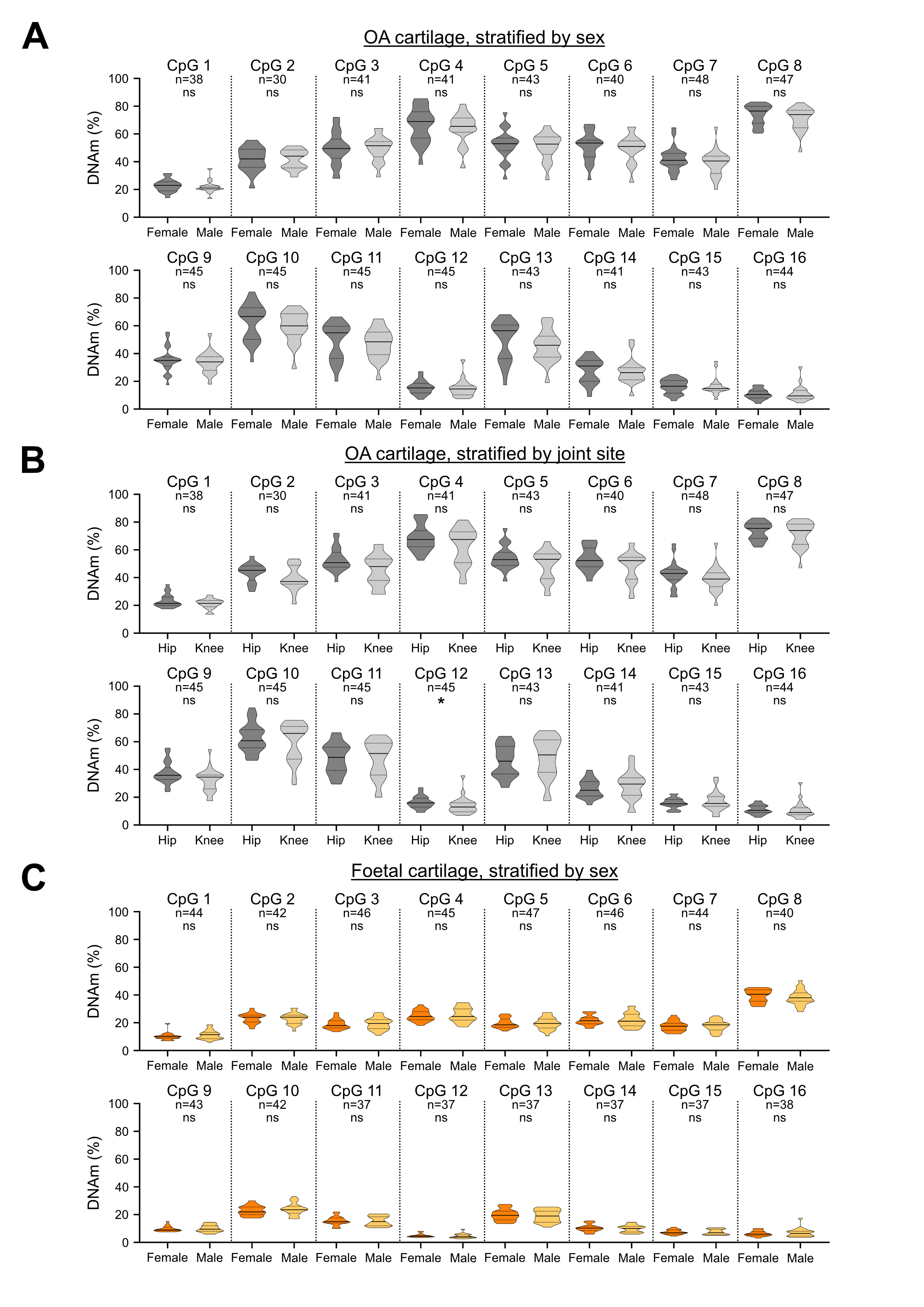

Supplement: Supplementary file 8 — Supplementary Material 8 [file 13075_2024_3315_MOESM8_ESM.tiff]
